# The Use of Pain Severity and Its Impact as a Predictor for MRI Findings: A Cross-Sectional Study in Ta'if, Saudi Arabia

**DOI:** 10.7759/cureus.45463

**Published:** 2023-09-18

**Authors:** Ahmad H Alharbi, Tala AlSindi, Albaraa F Ashoor, Abdulrahman M Almalki, Mansour I Aljabri, Murouj Almaghrabi, Ahmed M Alothman, Nizar A Almaghrabi, Adnan M Boubaker

**Affiliations:** 1 Department of Medicine and Surgery, College of Medicine, Umm Al-Qura University, Al-Abdia Main Campus, Makkah, SAU; 2 Department of Neurosurgery, King Faisal Specialist Hospital and Research Centre, Jeddah, SAU; 3 Department of Radiology, King Abdulaziz Hospital, Makkah, SAU; 4 Department of Neurological Surgery, King Abdulaziz Specialist Hospital, Ta'if, SAU

**Keywords:** mri, dallas pain questionnaire (dpq), magnetic resonant imaging, chronic low back pain (clbp), low-back pain (lbp)

## Abstract

Background

Low back pain (LBP) is common and considerably impacts daily lives across all age groups. MRI is not frequently used as a first-line investigation for patients presenting with LBP, except in the presence of red-flag symptoms. This study aimed to use pain severity and its impact as a predictor for MRI findings to help physicians decide whether a patient needs an MRI.

Methods

This cross-sectional study was conducted at the outpatient clinic of the neurosurgery department. The questionnaire included demographic data of the patients, red-flag symptoms, and the Dallas Pain Questionnaire (DPQ). The primary physician then determines whether the patient should have an MRI appointment.

Results

The study included 100 patients with LBP, of which 71 had chronic LBP (CLBP). Out of these 71, an MRI was requested for 62, but only 26 had findings related to LBP. Regarding the impact of CLBP on daily activities as measured by the DPQ, there was a significant association between those whose CLBP affected their daily activities and the decision to request an MRI. However, no significant statistical association was found between the three other parameters of the DPQ and the primary physician's decision to request an MRI.

Conclusion

Concerning the use of the DPQ questionnaire to predict MRI findings in patients with CLBP, the study indicates that significant pain impact on the DPQ does not necessarily correlate with MRI findings related to LBP. This suggests that the DPQ evaluation tool has no advantage over a physician's clinical judgment.

## Introduction

Low back pain (LBP) is a common condition that considerably impacts daily life activities in all ages [1]. In 2015, it was established that 540 million individuals were affected by LBP at some point [2]. A 2020 systematic review of five studies revealed that the prevalence of LBP within specific occupational disciplines in Saudi Arabia was between 64% and 84%. However, the incidence of LBP among the general population in the Kingdom of Saudi Arabia remains somewhat unclear [3-4]. Patients with LBP can present in either an acute form, with 90% resolving spontaneously within 12 weeks, or a chronic form. Common causes of LBP can be categorized as mechanical (e.g., disc herniation, spinal stenosis, strain, sprain, spondylolysis, fracture, and trauma), nonmechanical due to a compressive mass or inflammation, and non-specific LBP, which represents 95% of LBP cases [5]. Compared to CT scans and X-rays, MRI offers superior soft-tissue visualization without exposing the patient to radiation [4]. Yet, MRI is not typically the first-line investigation unless there are red flag signs and symptoms indicating a potential serious underlying pathology that warrants an urgent MRI. These red flags include a previous history of cancer, chronic steroid use, history of IV drug abuse, immunosuppression, HIV, weight loss, fever, chills, night sweats, pain that worsens when supine, bowel or bladder dysfunction, thoracic pain, saddle anesthesia, and radicular pain [4,6]. Nevertheless, some signs and symptoms are considered red flags in certain guidelines but not in others [7,8]. This study focuses on a specific clinical scenario commonly encountered by both general physicians and specialists. We utilized the Dallas Pain Questionnaire (DPQ) to assess the impact of chronic LBP on patients' daily activities, work and leisure, anxiety, depression, and social interests. The goal was to determine whether the overall impact of pain correlates with MRI findings.

## Materials and methods

Study design and selection of patients

This study is a descriptive cross-sectional design. The study used a convenience sampling technique to include patients with LBP who presented to the neurosurgical outpatients’ department (OPD), Taif, Saudi Arabia, between July 2021 to August 2021 (two months). Ethical approval was granted from the institutional review board (IRB) of the Directorate of Health Affairs - Taif (Approval no. HAP-02-T-067). The total number of neurosurgical clinics was two and managed by four neurosurgeon consultants. Exclusion criteria were patients below adolescent age (12 years) and patients who failed to attend their MRI appointments.

Data collection and assessment criteria

A printed, self-reported questionnaire was set up before the clinic appointment in the waiting area for data collection, which was gathered by nine independent data collectors. The collected parameters for all patients included age, sex, nationality, weight, height, comorbidities (hypertension and diabetes mellitus), duration of pain (acute, sub-chronic, chronic), detailed history of the presenting illness, associated symptoms, and general red flags of LBP (extremes of age (<20 or >55 years), cauda equina syndrome, sudden bladder dysfunction, progressive weakness, gait disturbance, sciatica, saddle (perineal) numbness, fecal incontinence, widespread sensory deficit in the lower limbs, pain radiating to the lower limbs). There were also questions regarding malignancy history (previous history of malignancy, unexplained weight loss, reduced appetite, rapid fatigue, fever), fractures, and infection history (history of major trauma, use of systemic steroids, previous bacterial infection, immunodeficiency, urinary tract infection, previous back surgery), and whether an MRI was requested by the clinic physician (neurosurgery's resident, specialist, or consultant) for the patient, independent of the questionnaire. The MRI findings of the patients were collected through the hospital imaging system, obtaining T1- and T2-weighted sagittal and axial images. An assigned expert radiologist interpreted all images to evaluate the significance of each image. Significant positive findings included cases with a disc bulge, herniation, or protrusion causing nerve compression or spinal stenosis.

For patients with chronic LBP, the Dallas Pain Questionnaire (DPQ) was used to assess the impact of pain [9]. The tool is a 16-item visual analog, which was developed and validated in many languages to evaluate patients’ perceptions of the effects of chronic pain on four aspects of their lives: (i) daily activities, including pain intensity, personal care, lifting, walking, sitting, standing, and sleeping; (ii) work and leisure activities, including social life, traveling, and vocation; (iii) anxiety-depression; and (iv) social interest, which includes interpersonal relationships, social support, and punishing responses. Since there is no available validated Arabic version of the DPQ, a back-to-back translation technique was used [10]. Two independent native Arabic doctors from the English department at Umm Al-Qura University, Makkah, Saudi Arabia, translated the original English version of the DPQ into Arabic. The Arabic version was then translated back into English by two independent native English physicians fluent in Arabic. A pilot study was conducted among 15 patients before the final collection of data.

Statistical analysis

After obtaining data from eligible patients for the study, data were cleaned, coded, and entered into the statistical software IBM SPSS version 23.0 for analysis. Data were analyzed using means ± SDs for quantitative variables, while frequencies and percentages were used for qualitative variables. To compare qualitative variables, a Chi-square test was performed. Means were compared using the ANOVA test. A p-value ≤ 0.05 was regarded as statistically significant. The duration of LBP was classified as acute pain (<6 weeks), sub-chronic pain (6-12 weeks), and chronic pain (>12 weeks) [11]. To interpret the results of the DPQ for patients with chronic LBP, each of the 16-item scales ranged from 0% on the left end (which means "no pain" or "not at all") to 100% on the right end (which means "all the time"). Each scale includes five to eight segments that assign a value from 0 to 7 (left end to right end, respectively). Each of the four aspects of the tool was calculated independently by summing the aspect's items and multiplying it by a constant. As follows: (i) daily activities: sum of items 1-7 * 3, (ii) work and leisure activities: sum of items 8-10 * 5, (iii) anxiety-depression: sum of items 11-13 * 5, (iv) social interest: sum of items 14-16 * 5. This calculation has provided a percentage reflecting the impact of LBP on the aspect being considered. Each aspect was then categorized into three groups according to the severity as follows: (i) mild impact (0-33%); (ii) moderate impact (34-66%); and (iii) great impact (67-100%) [12].

## Results

General characteristics of the included participants**

The current study included 100 patients with LBP. Table 1 presents the general characteristics of the patients, categorized by the duration of LBP. It includes data missing from 10 patients. Most of the patients had CLBP (71%). The mean age of the patients was 46.5 ± 15.9. More than half of the patients were female (60%) and Saudi nationals (96.7%). Nearly half of the patients had a history of chronic diseases (46.7%), including hypertension (27.8%) and diabetes mellitus (18.9%). Most patients had a BMI indicating obesity, followed by those in the overweight category (41% and 34.9%, respectively).

**Table 1:** General characteristics of the patients by the duration of low back pain. (-) Data related to the duration of LBP contain 10 missing pieces of information in regards to the duration of low back pain.

Variables	Acute (<6 weeks) (n=12)		Sub-chronic (6-12 weeks) (n=7)		Chronic (>12 weeks) (n=71)		Total (n=90)	
	No	%	No	%	No	%	No	%
Age (years) mean, SD	40.5 ± 17.9		43.1 ± 17.9		47.9 ± 15.3		46.5 ± 15.9	
Sex								
Male	6	50.0%	4	57.1%	26	36.6%	36	40.0%
Female	6	50.0%	3	42.9%	45	63.4%	54	60.0%
Nationality								
Saudi	12	100%	7	100%	68	95.8%	87	96.7%
Non-Saudi	0	-	0	-	3	4.2%	3	3.3%
Comorbidities								
Hypertension	4	33.3%	1	14.3%	20	28.2%	25	27.8%
Diabetes mellitus	0	-	1	14.3%	16	22.5%	17	18.9%
Smoking status								
Non-smokers	11	91.7%	6	85.7%	65	91.5%	82	91.1%
Current smokers	1	8.3%	1	14.3%	6	8.5%	8	8.9%
BMI								
Underweight (<18.5)	1	9.1%	0	-	2	3.1%	3	3.6%
Normal range (18.5-24.9)	3	27.3%	4	57.1%	10	15.4%	17	20.5%
Overweight (25.0-29.9)	5	45.5%	0	-	21	32.3%	29	34.9%
Obese (≥30.0)	2	18.2%	3	42.9%	32	49.2%	34	41.0%

**Figure 1:**
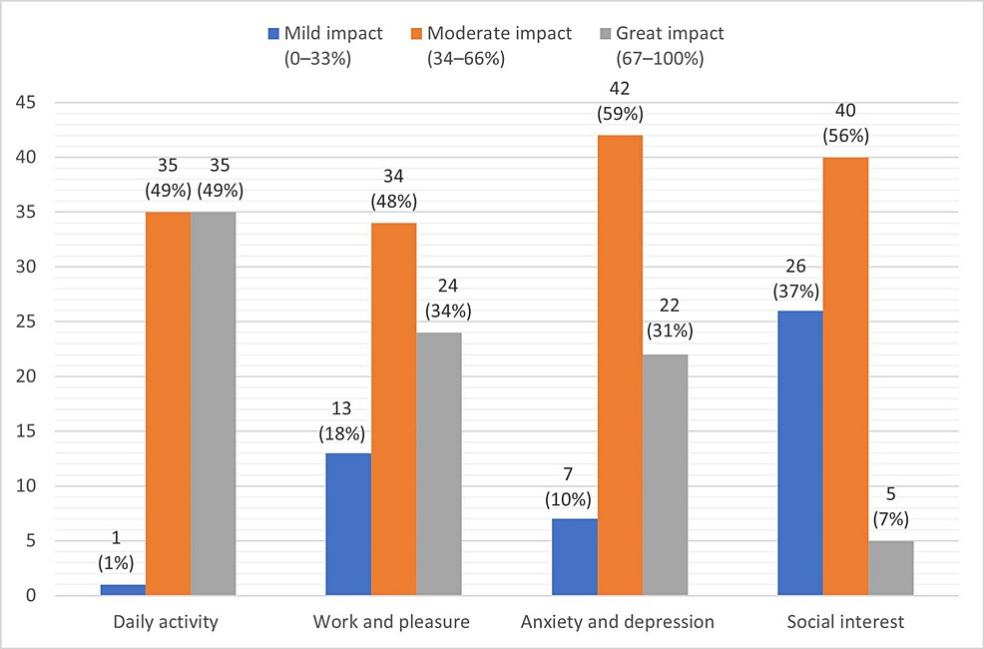
The impact of pain among patients with chronic LBP using DPQ (n=71). LBP: Lower back pain; DPQ: Dallas Pain Questionnaire.

The impact of pain among patients with CLBP was assessed using the DPQ (Figure 1) in all four domains: daily activity, work and pleasure, anxiety and depression, and social interest. The moderate impact of pain was highly registered among the participants in all four domains (49%, 48%, 59%, and 56%, respectively). The greatest impact of pain was reported for daily activity (48%) and work and pleasure (34%).

Features of MRI status and findings among patients with LBP

**Figure 2:**
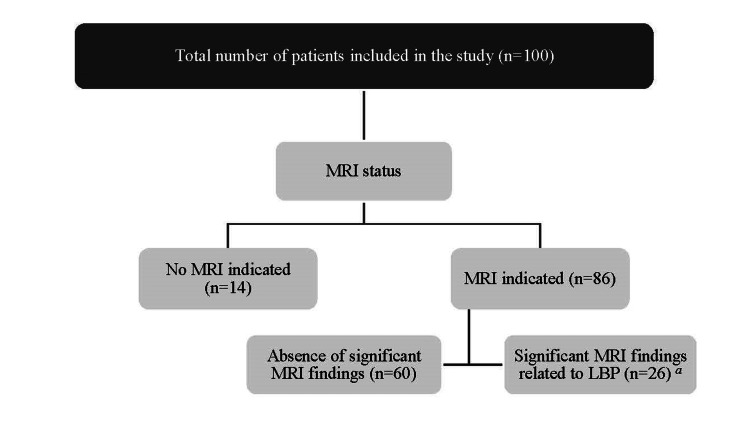
A concept map of the MRI status and its findings among all patients (n=100). a: MRI findings detected evidence of LBP by bulging, herniation, protrusion, or degeneration that causes nerve compression or spinal stenosis. LBP: Low back pain.

Almost all of the patients who presented to the clinic with LBP had an MRI spine requested by the physician (86 patients). From those whom an MRI was requested for, only 26 patients had findings related to LBP, such as bulging, herniation, protrusion, or degeneration that causes nerve compression or spinal stenosis. In comparison, 60 patients had no findings related to LBP. Additional information is shown in the concept map (Figure 2). The association between MRI status and red flags of LBP is presented in Table 2. Hence, MRI was requested for most patients with general red flags (p = 0.03). In addition, patients with fewer red flags (1-2) had not requested an MRI (50%), while more recorded red flags (3-5) were more likely to request an MRI (46.5%) (p = 0.02).

**Table 2:** Patients’ MRI status in association with LBP red flags (n=100). * Independent sample t-test or Pearson chi-square test as appropriate. The difference is considered significant at a p-value ≤ 0.05. LBP: Low back pain.

Variables	MRI status of the patients				P-value
	MRI requested (n=86)		No MRI needed (n=14)		
Age (n, %)					
Extreme ages (<20 or >55 years)	34	39.5%	7	50.0%	0.46
General red flags of LBP (n, %)					
Presence of at least one risk factor	76	88.4%	8	57.1%	0.03*
None	10	11.6%	6	42.9%	
Malignancy history/symptoms (n, %)					
Presence of at least one risk factor	56	65.1%	6	42.9%	0.11
None	30	34.9%	8	57.1%	
Fractures and infection history (n, %)					
Presence of at least one risk factor	39	45.3%	6	57.1%	0.87
None	47	54.7%	8	42.9%	
Total number of red flags (n, %)					
No present red flags	2	2.3%	2	14.3%	0.02*
1-2 red flag/s	22	25.6%	7	50.0%	
3-5 red flags	40	46.5%	4	28.6%	
>5 red flags	22	25.6%	1	7.1%	

Table 3 reveals the factors associated with MRI findings among patients with LBP. While the reporting of red flags has increased the request for MRI, it has no association with the presence of significant findings related to LBP in the MRI results.

**Table 3:** Factors associated with MRI findings among all patients with LBP. * Independent sample t-tests were used. The difference is considered significant at a p-value ≤ 0.05. LBP: Low back pain.

Variables	MRI findings of the patients		
	MRI findings related to LBP ^a^	MRI findings not related to LBP	P-value*
Presence of any general red flags	24 (29.6%)	57 (70.4%)	0.624
Presence of any malignancy red flags	18 (32.1%)	38 (67.9%)	0.598
Presence of any fracture/infection red flags	9 (23.1%)	30 (76.9%)	0188
Total number of red flags			
No red flags	2 (100%)	(0.0)	0.052
1-2 red flags	5 (22.7%)	17 (77.3%)	
3-5 red flags	15 (37.5%)	25 (62.5%)	
> 5 red flags	4 (18.2%)	18 (81.8%)	
Diagnosed with any psychological illness	6 (35.3%)	11 (64.7%)	0.612
Diagnosed with any comorbidities	11 (42.3%)	15 (57.7%)	0.108
Smoking status			
Current/previous smoker	1 (14.3%)	6 (85.7%)	0.338
Non-smoker	25 (31.6%)	54 (68.4%)	
BMI			
Underweight (<18.5)	0 (0.0)	1 (100%)	0.936
Normal weight (18.5-24.9)	4 (26.7%)	11 (73.3%)	
Overweight (25.0-29.9)	9 (28.1%)	23 (71.9%)	
Obese (>30.0)	9 (29.0%)	22 (71.0%)	

DPQ in association with other variables

The impact of LBP was assessed in association with the decision of the MRI request. Regarding the impact on daily activity, most patients who did not request an MRI had a moderate impact. In contrast, MRI was mostly requested among patients with a great impact (55%), followed by moderate impact (44%) with a statical significance association of 0.03. Additional information on the impact on other domains is presented in Table 4.

**Table 4:** The association between DPQ scores and MRI requests among patients with CLBP (n=71). * Cross-tabulation was used. The difference is considered significant at a p-value ≤ 0.05. CLBP: Chronic low back pain; DPQ: Dallas Pain Questionnaire.

DPQ of pain impact	MRI status of the patients		P-value^*^
	MRI requested Total: 62 (87%)	No MRI requested Total: 9 (13%)	
Effect on daily activity			
Mild impact	1 (1%)	0 (0.0%)	0.03*
Moderate impact	27 (44%)	8 (89%)	
Great impact	34 (55%)	1 (11%)	
Effect on work and leisure			
Mild impact	12 (19%)	1 (11%)	0.48
Moderate impact	28 (45%)	6 (67%)	
Great impact	22 (36%)	2 (22%)	
Effect on anxiety-depression			
Mild impact	5 (8%)	2 (22%)	0.22
Moderate impact	36 (58%)	6 (67%)	
Great impact	21 (34%)	1 (11%)	
Effect on social interest			
Mild impact	21 (34%)	5 (562%)	0.37
Moderate impact	36 (58%)	4 (44%)	
Great impact	5 (8%)	0 (0.0%)	

Table 5 revealed the association between DPQ scores and MRI findings. Hence, MRI findings related to LBP were shown among patients with moderate and great impact on daily activity (50% of each).

**Table 5:** The association between DPQ scores and MRI findings among patients with CLBP. * Cross-tabulation was used. The difference is considered significant at a p-value ≤ 0.05. £ MRI findings detected evidence of LBP by bulging, herniation, protrusion, or degeneration (disc, facet, ligament) that causes nerve compression or spinal stenosis. LBP: Low back pain; DPQ: Dallas Pain Questionnaire; CLBP: Chronic low back pain.

DPQ of pain impact	MRI findings of the patients		P-value^*^
	MRI findings related to LBP (18) ^£ ^	MRI findings not related to LBP (44)	
Effect on daily activity			
Mild impact	0 (0.0)	1 (2.3%)	0.684
Moderate impact	9 (50.0%)	18 (40.9%%)	
Great impact	9 (50.0%)	25 (56.8%)	
Effect on work and leisure			
Mild impact	3 (16.7)	9 (20.5%)	0.570
Moderate impact	10 (55.6%)	18 (40.9%)	
Great impact	5 (27.8%)	17 (38.6%)	
Effect on anxiety-depression			
Mild impact	1 (5.6%)	4 (9.1%)	0.517
Moderate impact	9 (50.0%)	27 (61.4%)	
Great impact	8 (44.4%)	13 (29.5%)	
Effect on social interest			
Mild impact	4 (22.2%)	17 (38.6%)	0.442
Moderate impact	12 (66.7%)	24 (54.5%)	
Great impact	2 (11.1%)	3 (6.8%)	

The total number of red flags concerning DPQ scores is shown in Table 6. Most patients with a great impact of pain on daily activity had between three and five red flags (54.3%), followed by more than five red flags (25.7%). Furthermore, similar results were reported on the effect of pain on work and leisure, where most patients with a significant impact of pain had three to five red flags (50%) and more than five red flags (29.2%). Regarding the impact of pain on anxiety and depression, an equal number of patients had three to five red flags and more than five red flags (40.9% for each group). The effect on the social interest domain was the least affected by the number of red flags among patients with LBP.

**Table 6:** The association between DPQ scores and the total number of red flags among patients with CLBP (n=71). * Cross-tabulation was used. The difference is considered significant at a p-value ≤ 0.05. LBP: Low back pain; DPQ: Dallas Pain Questionnaire; CLBP: Chronic low back pain.

DPQ of pain impact	Total number of red flags of LBP				P-value^*^
	No red flags	1-2 red flags	3-5 red flags	> 5 red flags	
Effect on daily activity					
Mild impact	0 (0.0)	0 (0.0)	1 (100%)	0 (0.0)	0.22
Moderate impact	3 (8.6%)	12 (34.3%)	9 (25.7%)	11 (31.4%)	
Great impact	1 (2.9%)	6 (17.1%)	19 (54.3%)	9 (25.7%)	
Effect on work and leisure					
Mild impact	1 (7.7%)	6 (46.2%)	2 (15.4%)	4 (30.8%)	0.44
Moderate impact	2 (5.9%)	8 (23.5%)	15 (44.1%)	9 (26.5%)	
Great impact	1 (4.2%)	4 (16.7%)	12 (50.0%)	7 (29.2%)	
Effect on anxiety-depression					
Mild impact	1 (14.3%)	4 (57.1%)	1 (14.3%)	1 (14.3%)	0.21
Moderate impact	2 (4.8%)	11 (26.2%)	19 (45.2%)	10 (23.8%)	
Great impact	1 (4.5%)	3 (13.6%)	9 (40.9%)	9 (40.9%)	
Effect on social interest					
Mild impact	3 (11.5%)	9 (34.6%)	10 (38.5%)	4 (15.4%)	0.12
Moderate impact	0 (0.0%)	8 (20.0%)	18 (45.0%)	14 (35.0%)	
Great impact	1 (20.0%)	1 (20.0%)	1 (20.0%)	2 (40.0%)	

## Discussion

This study attempted to use the DPQ questionnaire to assist physicians in assessing a patient's disability without MRI indications to determine the need for an MRI, especially in the working-age group. However, the results did not demonstrate the superiority of the DPQ evaluation tool over physician judgment. Since recent guidelines recommend against routine MRI imaging in the absence of red flags, further studies with larger sample sizes are needed to assess the DPQ tool as a predictor for MRI findings in patients with CLBP [7,8]. The results revealed that of the 62 patients with chronic low back pain who visited the clinic and for whom the physician requested an MRI, 34 (55%) reported a significant impact of pain on their daily activities, and 22 (36%) felt a pronounced impact of pain on their work and leisure (Table 4). However, only 18 (28%) patients exhibited MRI findings related to LBP (Table 5). Consistent with the current results, a previous study demonstrated that in most countries, LBP is the leading cause of disability [2], especially within the working age group [2,1]. This observation can likely be attributed to the fact that non-specific low back pain is the most prevalent form [5,13]. The pathogenesis of non-specific back pain remains elusive. Some studies suggest that factors such as genetics, psychological status, smoking habits, obesity, and work-related behaviors might be influential [14]. In such cases, an MRI image is not sufficient for diagnosing this type of LBP.

The study was not limited to a specific age group. The mean age of the participants was 46.5 ± 15.9 years, with a female predominance. A systematic review study conducted in 2020 included five cross-sectional studies that investigated the prevalence and characteristics of LBP in Saudi Arabia. These studies showed that all targeted the working-age group with a mean age of 33.9-38 years [3]. In contrast, a community-based study conducted a year later in Riyadh on individuals with LBP was limited to those 40 years old or older (mean age 50.78 ± 8.15 years) and also demonstrated a female predominance [15]. A global systematic review that included only population-based studies concluded that the prevalence of CLBP among individuals > 50 years old is two to three times higher than in those under 30, with a consistent female predominance [16]. A meta-analysis of observational studies conducted in 2010 demonstrated a strong relationship between obesity/overweight and LBP, with an even stronger association with chronic LBP [17]. This finding aligns with the BMI status of our participants, of whom 75% are overweight or obese (Table 1). With regard to smoking status, the incidence of LBP is higher among smokers (OR 1·30 (1·16-1·45)) [18]. Surprisingly, the study results indicated that 91% of the participants were non-smokers. There was no significant association between MRI findings related to LBP and other variables (Table 3). This finding was also reported by a systematic review conducted in 2014, in which there was no clear association between the MRI findings and the clinical outcome. This might be because of the small sample size of the included studies with different populations and measures, which made it difficult to draw a clear conclusion from this systematic review [19].

Strength and limitations

This study is the first to attempt and utilize the DPQ as an assessment tool to enhance the physician's judgment in making a clearer MRI decision. Nevertheless, there are multiple limitations in the study, starting with the small number of participants (100 participants), with 71 having chronic back pain. The small sample size is because of the short window for data collection (two months), which was further complicated by restrictions related to the COVID-19 pandemic. Furthermore, the study was conducted in only one center in the region. Finally, a major limitation of the study was the funding and the inability to order lumbar MRI for patients determined by the primary physician as non-MRI patients; this can directly affect the results.

## Conclusions

Regarding using the DPQ questionnaire to predict MRI findings in patients with CLBP, the study indicates that a significant impact of pain in the DPQ tool does not necessarily correspond to findings in MRI imaging related to LBP. This suggests that the DPQ evaluation tool does not hold superiority over a physician's clinical judgment. Current guidelines advise against routine MRI imaging in the absence of red flags. Therefore, further research with larger sample sizes is recommended. Additionally, while the DPQ tool was employed to assess the impact of LBP in this study, exploring other tools that might predict the need for an MRI is suggested.

## References

[1] Hartvigsen J, Hancock MJ, Kongsted A (2018). What low back pain is and why we need to pay attention. Lancet.

[2] BD 2015 Disease and Injury Incidence and Prevalence Collaborators (2016). Global, regional, and national incidence, prevalence, and years lived with disability for 310 diseases and injuries, 1990-2015: a systematic analysis for the Global Burden of Disease Study 2015. Lancet.

[3] Aldera MA, Alexander CM, McGregor AH (2020). Prevalence and Incidence of low back pain in the Kingdom of Saudi Arabia: a systematic review. J Epidemiol Glob Health.

[4] Selkirk SM, Ruff R (2016). Low back pain, radiculopathy. Handb Clin Neurol.

[5] Wilk V (2004). Acute low back pain: assessment and management. Aust Fam Physician.

[6] Delitto A, George SZ, Van Dillen L (2012). Low back pain. J Orthop Sports Phys Ther.

[7] Oliveira CB, Maher CG, Pinto RZ (2018). Clinical practice guidelines for the management of non-specific low back pain in primary care: an updated overview. Eur Spine J.

[8] Corp N, Mansell G, Stynes S, Wynne-Jones G, Morsø L, Hill JC, van der Windt DA (2021). Evidence-based treatment recommendations for neck and low back pain across Europe: a systematic review of guidelines. Eur J Pain.

[9] Lawlis GF, Cuencas R, Selby D, McCoy CE (1989). The development of the Dallas Pain Questionnaire. An assessment of the impact of spinal pain on behavior. Spine (Phila Pa 1976).

[10] Ang BH, Chen WS, Ngin CK, Oxley JA, Lee SW (2018). Reliability and validity of the English and Malay versions of the Driving and Riding Questionnaire: a pilot study amongst older car drivers and motorcycle riders. Public Health.

[11] Koes BW, van Tulder M, Lin CW, Macedo LG, McAuley J, Maher C (2010). An updated overview of clinical guidelines for the management of non-specific low back pain in primary care. Eur Spine J.

[12] Marty M, Rozenberg S, Duplan B, Thomas P, Duquesnoy B, Allaert F (2008). Quality of sleep in patients with chronic low back pain: a case-control study. Eur Spine J.

[13] Maher C, Underwood M, Buchbinder R (2017). Non-specific low back pain. Lancet.

[14] Balagué F, Mannion AF, Pellisé F, Cedraschi C (2012). Non-specific low back pain. Lancet.

[15] Alhowimel AS, Alodaibi F, Alshehri MM, Alqahtani BA, Alotaibi M, Alenazi AM (2021). Prevalence and risk factors associated with low back pain in the Saudi adult community: a cross-sectional study. Int J Environ Res Public Health.

[16] Meucci RD, Fassa AG, Faria NM (2015). Prevalence of chronic low back pain: systematic review. Rev Saude Publica.

[17] Shiri R, Karppinen J, Leino-Arjas P, Solovieva S, Viikari-Juntura E (2010). The association between obesity and low back pain: a meta-analysis. Am J Epidemiol.

[18] Shiri R, Karppinen J, Leino-Arjas P, Solovieva S, Viikari-Juntura E (2010). The association between smoking and low back pain: a meta-analysis. Am J Med.

[19] Steffens D, Hancock MJ, Maher CG, Williams C, Jensen TS, Latimer J (2014). Does magnetic resonance imaging predict future low back pain? A systematic review. Eur J Pain.

